# Enhanced Skin Penetration of Cannabidiol Using Organosilane Particles as Transdermal Delivery Vehicles

**DOI:** 10.3390/pharmaceutics15030798

**Published:** 2023-02-28

**Authors:** Zahra Khabir, Connie Partalis, Jimit Vijay Panchal, Anand Deva, Aparajita Khatri, Alfonso Garcia-Bennett

**Affiliations:** 1 Australian Research Council, Industrial Transformation Training Centre for Facilitated, Advancement of Australia’s Bioactives (FAAB), Macquarie University, Sydney, NSW 2109, Australia; 2 School of Natural Sciences, Macquarie University, Sydney, NSW 2109, Australia; 3 Faculty of Medicine, Health and Human Sciences, Macquarie University, Sydney, NSW 2109, Australia; 4 EncapSolutions Pty. Ltd., 11 Julius Avenue, North Ryde, NSW 2113, Australia

**Keywords:** transdermal release, cannabidiol, nanoporous drug delivery, slow-release formulations

## Abstract

There is potential for cannabidiol to act as an analgesic, anxiolytic and antipsychotic active ingredient; however, there is a need to find alternate administration routes to overcome its low oral bioavailability. In this work, we propose a new delivery vehicle based on encapsulation of cannabidiol within organosilica particles as drug delivery vehicles, which are subsequently incorporated within polyvinyl alcohol films. We investigated the long-term stability of the encapsulated cannabidiol, as well as its release rate, in a range of simulated fluids with different characterization techniques, including Fourier Transform Infrared (FT-IR) and High-performance Liquid Chromatography (HPLC). Finally, we determined the transdermal penetration in an ex vivo skin model. Our results show that cannabidiol is stable for up to 14 weeks within polyvinyl alcohol films at a range of temperatures and humidity. Release profiles are first-order, consistent with a mechanism involving diffusion of the cannabidiol (CBD) out of the silica matrix. The silica particles do not penetrate beyond the stratum corneum in the skin. However, cannabidiol penetration is enhanced and is detected in the lower epidermis, which was 0.41% of the total CBD in a PVA formulation compared with 0.27% for pure CBD. This is partly due to an improvement of its solubility profile as it is released from the silica particles, but we cannot rule out effects of the polyvinyl alcohol. Our design opens a route for new membrane technologies for cannabidiol and other cannabinoid products, where administration via non-oral or pulmonary routes can lead to better outcomes for patient cohorts in a range of therapeutics.

## 1. Introduction

**Scheme 1:**
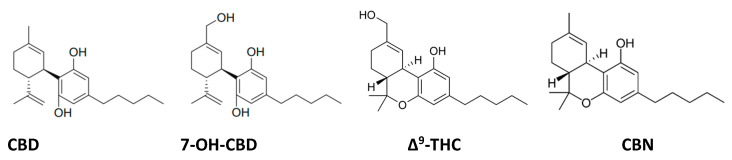
Cannabidiol (CBD) and its primary metabolite in the liver 7-hydroxycannabidiol (7-OH-CBD). CBD can degrade by cyclization under acidic conditions to Δ⁹-tetrahydrocannabinol (Δ⁹-THC) and eventually to cannabinol (CBN) by oxidation.

Cannabidiol (CBD) is a non-psychoactive cannabinoid product with few negative side effects. At low doses it possesses antioxidative, anti-inflammatory and neuroprotective effects [1,2], and it has been recently reported for the treatment of glaucoma [3]. This has led to an increase in the number of clinical trials registered to exploit the uses of CBD, with over 500 currently listed on clinicaltrials.gov (accessed on 1 October 2022) [4]. While there is clinical evidence of the use of ingestible cannabinol (mainly a combination of tetrahydrocannabinol and CBD with an unspecified mix of other cannabinoids), there is a startling lack of scientific validation of alternative administration routes that can potentially avoid formulation problems associated with the poor solubility profile of CBD and first-pass metabolism by cytochrome P450 enzymes in the liver, and the subsequent formation of the CBD metabolite 7-OH-CBD (Scheme 1) [5]. The absolute bioavailability of CBD after oral dosing, under fasting conditions, is approximately 6% [6]. It is reported to increase fourfold when the medication is co-administered with a high-fat meal; however, these values remain relatively low considering that therapeutic levels of CBD are in a dose range as high as 1000 mg/day [7]. There is thus a need to develop alternative formulations and administration routes to improve the bioavailability and efficacy of CBD treatments.

Whilst most clinical research is focused on oral and pulmonary administration routes [8], there are some early studies on other formulation strategies. Intravenous administration of 20 mg of deuterium-labelled CBD achieved mean plasma CBD concentrations of 686 ng/mL (3 min post-administration), which very rapidly dropped to 48 ng/mL after 1 h [9]. Unfortunately, there is a lack of in vivo transdermal delivery data [10]. Giacoppo et al. have reported a CBD formulation at a 1% dose, prepared in a dense cream containing propylene glycol, showing therapeutic effects in a mouse model of experimental autoimmune encephalomyelitis [11]. The maximum plasma concentration (C_max_) achieved with an application area of 1 cm^2^ was 8.3  ±  2.1 ng/mL. Hammell et al. have prepared a topical CBD gel containing Carbopol 980 polymer, a crosslinked polyacrylic acid that is polymerized in a sodium hydroxide solution [12]. Doses of 6.2 mg/day achieved plasma concentrations of 33.3 ng/mL in rats, showing relief of arthritis pain in a rat complete Freund’s adjuvant-induced monoarthritis knee joint model. It is important to note that both these studies utilize highly concentrated oil-in-ethanol creams. Ethanol is well known as a dermal penetration enhancer that can be used to increase the solubility of active compounds and decrease the dermal barrier, thus increasing permeability. However, while offering additional stability to aqueous formulations of CBD, ethanolic solutions can considerably degrade CBD, which is also sensitive to oxidation and photolytic reactions [13,14]. Understanding CBD’s degradation mechanism and kinetics in topical formulations under various temperatures and pH is important to its clinical use and storage. There are numerous reports detailing the degradation of CBD through cyclization reactions to the psychoactive compound Δ⁹-tetrahydrocannabinol(Δ^9^-THC), and subsequently by oxidation reaction to cannabinol (CBN) [15], and therefore, this is an important factor for any therapeutic use of CBD.

Herein, we develop a CBD formulation, based on its direct encapsulation within nanoporous organosilica particles, to improve the solubility profile of CBD in the absence of ethanol enhancers [16,17]. We prepare CBD-silica particles for dermal delivery using polyvinyl alcohol (PVA). As a water-soluble, stable polymeric cast, PVA can be moulded into a thin film and applied onto the skin [18]. In vitro and ex vivo experiments using human skin are carried out to evaluate the transdermal CBD delivery properties of the resulting stable polymeric films.

## 2. Materials and Methods

Organosilica particles, fluorescently labelled organosilica particles (FITC-silica) and CBD-loaded organosilica particles (CBD-Silica) received as a slurry were kindly provided by EncapSolutions Pty Ltd. (Sydney, Australia). Particles were kept in opaque sample containers and stored under nitrogen at 4 °C, until used. All other reagents were purchased from Sigma Aldrich and used as received without further purification unless stated otherwise. Prior to characterization, the slurry sample (CBD-Silica) was processed to obtain “dried”, “calcined” and “extracted” CBD-Silica samples. The dried sample was produced by snap-freezing the organosilica particles in liquid nitrogen and lyophilizing to sublime the water overnight. The calcined sample was prepared by heating organosilane particles to 550 °C with a ramp rate of 1 °C/min under airflow and holding at 550 °C for 220 min. The extracted sample refers to CBD-extracted organosilica particles. The extraction was performed by dispersion of organosilica particles in ethanol, stirring the mixture overnight and then centrifugation at 12,000 rpm for 10 min. After removing the supernatant, the remaining organosilica particles were collected and washed twice with ethanol. Finally, the sample was dried at 30 °C oven temperature overnight [19].

### 2.1. Synthesis of Organosilica Particles and CBD Loading

Organosilica CBD carriers were prepared by Encapsolutions Ltd. Pty (Sydney, Australia), as previously described and characterized [20,21]. Briefly, the synthesis was conducted by polymerization of silica precursors inside oil-in-water microemulsion droplets containing the active CBD or FITC. The microemulsion droplets act as mini reactors for inorganic polymerization of silica (tetraethyl orthosilicate, aminopropyl triethoxysilane and triethoxy(phenyl) silane).

### 2.2. PVA Casting

Casted mats were prepared in distilled water and PVA mixed at a ratio of 1:8 (6.7 g PVA to 54 mL water). Briefly, water is heated at 100 °C and the PVA is added slowly under vigorous stirring. Before the addition of particles, the heating is stopped, and the CBD-Silica particles are added (7.11 g). The mixture is stirred until homogenous, sonicated thoroughly for 20 min and then poured into a non-stick Teflon container with a diameter of 5 cm. The PVA-CBD-Silica is slowly dried at 30 °C in an oven overnight.

### 2.3. Characterization Techniques

#### 2.3.1. X-ray Diffraction (XRD) Analysis

XRD measurements were performed on the dried, calcined and extracted samples using a Bruker D8 Discover diffractometer equipped with Cu-Kα radiation as an X-ray source (λ = 1.5406 Å) working on a reflection mode. The measurement was carried out at the angular range of 0.5–70 (2θ) and 0.02 step size.

#### 2.3.2. Thermogravimetric Analysis (TGA)

Thermal stability and CBD loading of silica particles were assessed using a TGA instrument (Netzsch STA 449 F3 Jupiter-Gerätebau GmbH, Bavaria, Germany). Typically, 5 mg of slurry and dried samples were weighed and placed in ceramic crucibles and heated gradually from 25 °C to 900 °C at a 10 °C/min rate under an oxygen flow rate of 20 mL/min. The derivative weight loss was evaluated using Netzsch Proteus software (version 6.1.0).

#### 2.3.3. Nitrogen (N_2_) Adsorption Isotherm

The textural properties of silica particles, such as surface pore volume and pore size, were measured by the nitrogen adsorption/desorption isotherm technique with a TriStar II volumetric adsorption analyser (Micromeritics Instrument Corporation, Norcross, GA, USA). Prior to analysis, dried, calcined, extracted and slurry CBD-Silica particles were degassed with a sample degas system (Micromeritics VacPrep 061) overnight at room temperature. The average pore sizes and the surface area of the samples were calculated with the Barrett–Joyner–Halenda (BJH) and Brunauer–Emmett–Teller (BET) methods, respectively.

#### 2.3.4. Scanning Electron Microscopy (SEM)

The particle size and morphology of dried, calcined and extracted samples were studied using a JEOL JSM 7100F field emission scanning electron microscope (Tokyo, Japan) with a resolution of 1.2 nm, operating at 1.5 kV and without any gold coatings.

#### 2.3.5. Fourier Transform Infrared (FT-IR)

The functional groups of silica and CBD from dried, extracted and calcined samples were identified via a Nicolet iS5 FT-IR Spectrometer (Thermo Scientific, Waltham, MA, USA) with an iD5 ATR accessory in transmittance from 4000 cm^−^^1^ to 400 cm^−^^1^, and each measurement was averaged over multiple scans.

#### 2.3.6. Ultraviolet-Visible Spectroscopy (UV-Vis)

A Jasco V-760 UV/Vis spectrophotometer (Jasco, Tokyo, Japan) was employed to measure the UV absorption of slurry and dried CBD-Silica particles, as well as pure CBD, which was used as a reference. All samples were prepared in ethanol (100%) at a concentration of 0.08 mg/mL, and 210 nm wavelength was used for the UV absorption measurement.

#### 2.3.7. High-Performance Liquid Chromatography (HPLC)

The quantity of CBD released from slurry and dried CBD-Silica samples was determined with an Agilent 1260 Infinity II HPLC system (Agilent Technologies Inc., Santa Clara, CA, USA). Acetonitrile/water with 0.1% trifluoroacetic acid was used as mobile phase at a ratio of 75/25 (*v*/*v*) in an isocratic mode. A reverse-phase C18 column (5 μm, 250 × 4.6 mm; Grace Davison Discovery Sciences, Deerfield, IL, USA) was used, and the UV absorption was detected at 210 nm wavelength. The injection volume and flow rate were set at 20 μL and 0.8 mL/min, respectively. CBD calibration standards were prepared in ethanol (100%) at concentrations of 0.01, 0.05, 0.1, 0.5, 1, 5 and 10 μg/mL. Before the HPLC session, all samples were filtered through 0.2 μm nylon syringe filters (Whatman, Maidstone, UK).

#### 2.3.8. Dynamic Light Scattering (DLS)

The particle size, surface charge and level of agglomeration of slurry, dried and extracted CBD-Silica particles in aqueous solutions were evaluated by a Zetasizer ZS (Malvern Instruments, Worcestershire, UK) operating at a 173° detector angle at 25 °C and equipped with a 633 nm He-Ne laser. The particles were prepared at a concentration of 50 μg/mL in MilliQ water. All the samples were sonicated for 10 min before analysis.

#### 2.3.9. Inductively Coupled Plasma Mass Spectrometry (ICP-MS) and Elemental Microanalysis (CHN Analysis)

The concentrations of silicon (Si) released from silica particles in simulated gastrointestinal fluid (SGF) and MilliQ water media at a pH similar to simulated intestinal fluid (SIF) were measured using the ICP-MS technique. First, the collected samples were filtered (0.2 μm nylon syringe filter, Whatman) and then were analysed by an Agilent 7500 Quadrupole ICP-MS. CHN analysis was conducted on slurry, dried and extracted CBD-Silica particles to determine their molecular formula and compositional data, using an elemental analyser from Elementar (Vario El Cube, Berlin, Germany). Three aliquots of samples were weighed into boats and pressed, then combusted under O_2_. The percentages of carbon, hydrogen and nitrogen were calculated with the Dumas Method.

### 2.4. CBD Dissolution Experiments

The drug release profile was studied using Agilent Technologies 708-DS USP Apparatus under sink conditions connected to an Agilent Cary 60 UV-Vis spectrometer (Agilent Technologies, Santa Clara, CA, USA). The samples used for the dissolution studies were pure CBD, slurry CBD-Silica particles and PVA-CBD-Silica. The experiment ran for 72 h, and drug release was analysed using UV-Vis at periodic time intervals, under constant stirring at 50 rpm at 210 nm wavelength. To understand the release rate, the release profile was obtained for three different media, namely simulated sweat (SSW; pH 6.6), SIF (pH 6.8) and SGF (pH 1.2). For a more detailed description of the media compositions, see Table S1. The release of CBD in different media was evaluated using the Higuchi model.

### 2.5. CBD Degradation Studies

The degradation studies were performed using three different formulations that were kept in the dark all the time—pure CBD, slurry and PVA-CBD-Silica samples that were incubated at different temperatures (4 °C, 22 °C and 40 °C) with a humidity of 70%. The samples were collected in weeks 1, 2, 4 and 18. Afterwards, the collected samples were extracted with ethanol at 1 mg/mL and spun overnight before filtering (0.2 μm nylon syringe filter).

### 2.6. Ex Vivo Transdermal Studies

#### 2.6.1. Preparation of Excised Human Skin

A freshly excised, abdominal, human skin sample was received from a female donor (n = 1, aged 39 years) who had undergone abdominoplasty surgery. Before the research, an informed written consent was obtained from the participant (The Macquarie University Hospital, ethics approval protocol-5201200935). On the same day, the blood and subcutaneous fat were removed from the skin sample using a blunt dissection procedure. The skin sample was then stored aseptically at −30 °C and used within 6 months.

#### 2.6.2. In Vitro Skin Absorption Test

To determine the skin permeation of CBD, an in vitro skin absorption test was conducted using static Franz diffusion cells composed of donor and receptor chambers. Each Franz cell had an effective exposure area of 1.33 cm^2^, with a receptor volume of 3.5 mL. The absorption test was performed based on the OECD guidelines [22]. In short, defrosted skin samples were cut into disks and sandwiched between the donor and receptor compartments. Then, the donor and receptor of Franz cells were filled with sterile phosphate-buffered saline (PBS) and equilibrated at 32 °C for 30 min. To check the integrity of skin samples, their transepithelial electrical resistance (TEER) was measured with a multi-ohmmeter (Fluke, Everett, WA, USA), and samples with TEER < 20 kΩ were discarded. After TEER measurements, the PBS was removed, and the skin surface was blotted dry. Then, the receptor chamber was filled with media containing PBS: ethanol: Tween 20 (8.5:1:0.5), and three drops of skin surface fluid (a solution containing canola oil: SSW (1:1)) were dripped onto the skin surface [23]. The samples were then divided into four experimental groups in triplicates: (1) untreated skin (control) and skin samples treated with (2) PVA-CBD-Silica, (3) PVA-FITC-Silica and (4) pure CBD dissolved in ethanol. All samples were incubated for 72 h at 32 °C, and 500 μL of receptor fluid was sampled at predetermined times (0, 1, 5, 24, 48 and 72 h). At the end of the Franz cell test, the unabsorbed formulations were washed away with cotton swabs soaked in PBS, the Franz diffusion cells were then disassembled and the skin samples were removed. The collected skin samples, except the one treated with PVA-FITC-silica, were tape-striped (3M magic Scout tapes, 20 times) to remove most of the stratum corneum (SC) layer, and the remaining skin was minced and weighed accordingly. The collected receptor fluid, cotton swabs, tapes and remaining skin samples were kept at −4 °C to be analysed by HPLC.

#### 2.6.3. CBD Extraction Process

Absorbed CBD in the samples, including the collected cotton swabs, tapes and remaining skin, was extracted by immersing the samples in ethanol (100%) and shaking them in a rotary mixer (ELMI Intelli-Mixer™ RM-2M) for 12 h at room temperature. The remaining skin samples were also sonicated for 1 h during the extraction process. The CBD content of the extracted samples was determined with the HPLC method described in Section 2.3.7.

#### 2.6.4. Laser Scanning Confocal Microscopy (LSCM) Imaging

The skin penetration of the FITC-silica particles released from the PVA-FITC-silica was visualized with LSCM imaging. The skin samples treated with PVA-CBD-Silica in 2.6.2 were first cut into small pieces and then embedded in optimal cutting temperature (OCT) media in cryomolds (Tissue-Tek, Sakura, Japan), snap frozen at −80 °C on dry ice and transverse cross-sectioned into 20 µm slices using a cryostat at −20 °C (Leica CM 1950, Victoria, Australia).The sectioned samples were mounted on poly-L-lysine-coated microscope slides (Menzel-Glaser, Braunschweig, Germany) and then covered with glass coverslips (No. 1, 22 × 22 mm^2^ MenzelGläser) using a mounting medium (ProLong™ Gold Antifade Mountant, Invitrogen™) in between and sealed. Subsequently, the microscopy slides were imaged by an Olympus Fluoview (FV3000) confocal laser scanning microscope equipped with a 60 × oil-immersion objective lens (UPLSAPO60XS2; W.D.: 0.3 mm, silicon oil) and 8.0 μs per pixel scanning speed. For FITC, the microscope was illuminated with a 488 nm laser, and the emission was collected at the 500–550 nm spectral range.

## 3. Results

### 3.1. Composition and Characteristics of CBD-Silica Particles

**Figure 1:**
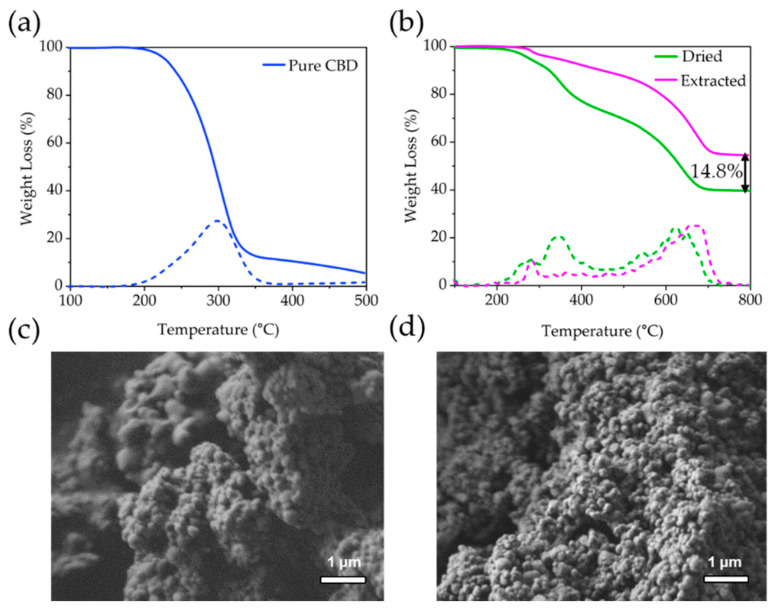
Thermogravimetric analysis profiles of (**a**) pure cannabidiol (CBD) and (**b**) CBD encapsulated within organosilane particles (CBD-Silica), after drying (Dried) and after extraction (Extracted) of the loaded CBD. Dotted lines correspond to the first derivative of the weight-loss curves shown. SEM images of CBD-Silica particle agglomerates before (**c**) and after (**d**) CBD extraction.

The XRD patterns collected from dry, calcined and extracted organosilane particles (Figure S1) show a main broad peak at 20° 2θ, which is characteristic of amorphous silica [24]. Samples of CBD-loaded organosilica particles (termed CBD-Silica) were received in the form of a slurry containing a high water content (20.5 wt%), as determined by TGA (see Supplementary Materials Figure S2). To assess the content of available CBD, samples were dried through a lyophilization step prior to extraction of the CBD in ethanol. Pure CBD shows a characteristic TGA weight-loss peak at 300 °C (Figure 1a) [25]. After drying, CBD-Silica shows 2 distinct weight-loss regions corresponding to desorption of propylamine groups at 220–320 °C (4.4 wt%) and phenyl groups at 500–700 °C (27.2 wt%), both from the organosilane matrix (Figure 1b). Decomposition of encapsulated CBD occurs at 300–420 °C, indicating a measured weight loss of 26.4 wt%. Approximately 14.8 wt% of CBD (56% of that loaded) was solvent extracted from the dried CBD-Silica sample after 12 h. There is no distinct change in the morphology of the silica particles after the extraction step, and they show large agglomerates with a primary particle size of 100 nm (Figure 1c,d).

**Figure 2:**
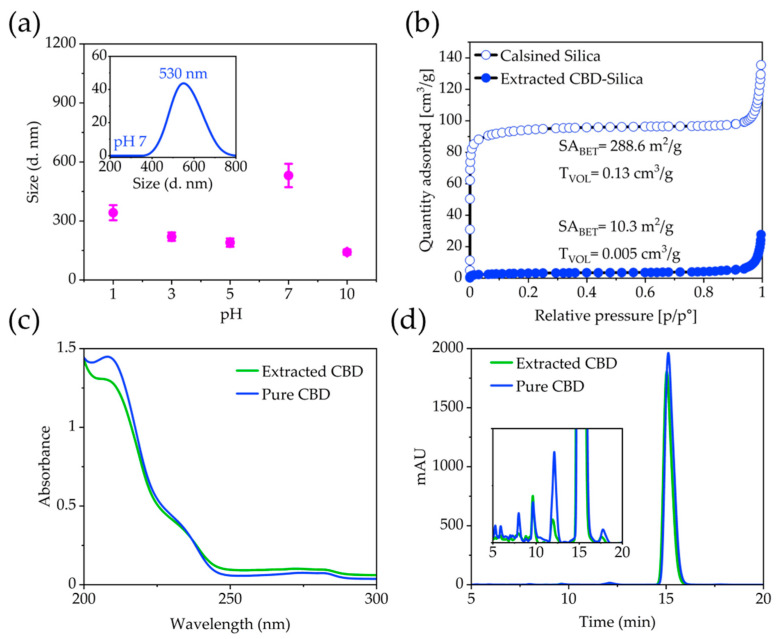
(**a**) DLS measurements conducted on CBD-Silica after 10 min sonication as a function of pH. Inset shows the measurement performed at pH 7. (**b**) N_2_ adsorption isotherms of extracted and calcined silica samples. Comparison of pure CBD and CBD extracted from silica. (**c**) UV-Vis spectra of pure and extracted CBD showing the characteristic absorption maximum at 210 nm. (**d**) Chromatograms of pure and extracted CBD showing a good correlation in the retention time.

**Figure 3:**
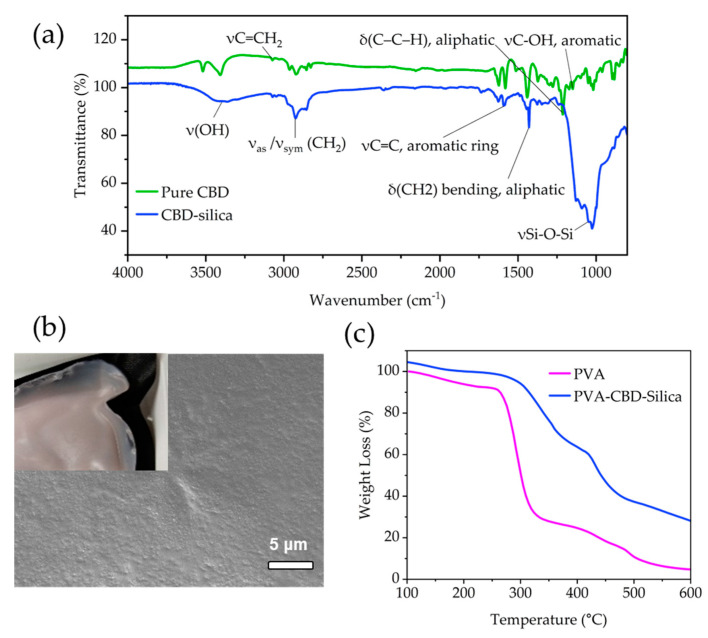
(**a**) FT-IR of pure CBD and CBD-silica particles. Tentative assignments for main stretching and peaks are included [27]. The small band at 3085–3060 cm^−1^ originates in the vinyl groups in CBD. (**b**) SEM images of the top surface of PVA-CBD-Silica film. Inset shows a macroscopic view of the film surface. (**c**) TGA profile of PVA-CBD-Silica film compared to PVA alone.

Dynamic light scattering (DLS) measurements show that the CBD-Silica particles remain within their agglomerated state, despite sonication in water at pH 7. The average intensity particle size distribution is centred around 550 nm (Figure 2a). Particles de-agglomerate under acidic and alkaline pH, with a size centred at 140 nm, measured at pH 10. Zeta potential measurements in water (not shown) reveal a net negatively charged surface with values of −14.8 mV for the CBD-Silica particles. No mesoporosity was observed in the dried CBD-Silica from N_2_ adsorption isotherms or after extraction of the CBD. However, when these were calcined at 550 °C in air to remove all organic content, a relatively high surface-area silica material was produced (288.6 m^2^/g, Figure 2b). Elemental microanalysis (CHN analysis) of the samples (Table S2) together with the TGA data allow calculating a molecular formula for the organosilane matrix of (C_6_H_5_SiO_1.5_)_0.514_(NH_2_(CH_2_)_3_SiO_1.5_)_0.172_(SiO_2_)_0.314_. To determine the stability of the extracted CBD, UV-Vis absorbance spectroscopy and high-performance liquid (HPLC) chromatography was used (Figure 2c,d). The results are consistent with previous reports for pure CBD, with a characteristic absorbance maximum at 210 nm, which was reproduced in the extracted CBD [26]. Similarly, chromatograms for pure and extracted CBD from the organosilica particles showed the same retention peaks, indicating that no detectable degradation of CBD occurred during the encapsulation or extraction process. The FT-IR spectra of the CBD encapsulated silica samples (Figure 3a) show similar vibration maxima to pure CBD, with the notable C = C aromatic stretching at 1580 cm^−1^ and the bending vibrations of CH_2_ and CH_3_ aliphatic groups between 1400–1200 cm^−1^ [27]. The presence of the band between 3085–3060 cm^−1^ owing to the vinyl group in pure CBD has been previously used for identification and quantification of CBD, and is observed in the encapsulated sample [28]. The strong Si-O-Si vibrational modes are clearly visible below ~1200 cm^−1^ [19,29].

A casting procedure in PVA was conducted to create a smooth thin-film (Figure 3b) of the CBD-silica particles, as described in the Materials and Methods section. The percentage loading of PVA was 26.7 wt% (Figure 3c), as determined from an average of 3 samples obtained from different regions of the film. The films turned a light pink colour, typical of exposure of CBD to light and oxygen [14].

**Figure 4:**
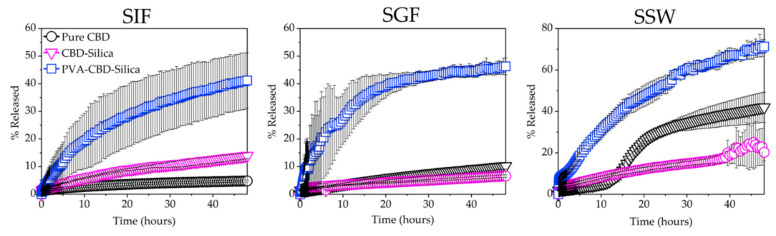
Dissolution profiles conducted at 30 °C in simulated intestine fluid (SIF, pH 6.8), simulated gastric fluid (SGF, pH 1.2) and simulated sweat (SSW, pH 6.6) under sink conditions for pure CBD, CBD-Silica and PVA-CBD-Silica film. Measurements are expressed as % release of total CBD in the formulation and were conducted in triplicates.

Dissolution experiments under sink conditions were conducted to measure the effects of PVA casting on the rate of release of CBD-Silica particles (Figure 4). For comparison, the dissolution profiles were measured in a range of simulated fluids at different pH, namely, simulated intestine fluid (SIF, pH 6.8), simulated gastric fluid (SGF, pH 1.2) and simulated sweat (SSW, pH 6.6). The last is used to mimic the transdermal environment, whilst SGF and SIF mimic oral administration and the gastrointestinal environment, respectively. As expected, pure CBD has a low solubility profile in both SIF and SGF, with less than 10% of CBD released in 48 h. These values are consistent with the solubility profile of CBD oil found by others [30]. In SSW, the solubility profile remains low in the first 24 h, but a jump is observed thereafter, with a total release in 48 h approximating 50% of the dose added. Since the pH in SSW and SIF is similar, this effect is presumably due to a component of the dissolution media in SSW, such as urea (Figure S3), which may enhance the solubility profile of CBD through complexation [31]. Further work is required to elucidate the mechanism of this change in solubility. The CBD-Silica samples behaved similarly in all media, with less than 25% released after 48 h, with the highest release (23%) observed in SSW. The release curves can be fitted with the Higuchi equation describing Fickian drug diffusion, [32,33] with a rate constant k_H_ = 2.91 (coefficient of determination R^2^ = 0.945) in SSW. CBD-Silica casted in PVA films show a significantly enhanced dissolution profile for all media, with up to 71% released in SSW, k_H_ = 10.40 (R^2^ = 0.992). These values demonstrate a large enhancement in the solubility of CBD as released from the PVA films.

The overall faster release kinetics for the PVA-CBD-Silica sample can be explained due to a more dispersed and less aggregated arrangement of particles in the PVA film achieved from the casting procedure, which increases the overall solubility profile of the CBD. A faster release was observed over the first hour in SSW (Figure S3), indicating that the PVA film itself is more readily dissolved in simulated sweat. To determine the effect of the silica matrix on the solubility of CBD, Inductively Coupled Plasma Mass Spectrometry (ICP-MS) analysis was conducted on the remaining SGF media from the dissolution experiments. The value for the silica content in the remaining SGF solution is 0.167 ppm, considerably lower than for the dissolution of the silica matrix in water (pH 6.8) under the same conditions (1.555 ppm). These results are consistent with the dissolution behaviour of high surface area, mesoporous silica particles under biological media [34]. These values, however, are well below the expected full dissolution of the silica matrix in the dissolution bath volume, which would be equivalent to 14.3 ppm of Si in the media post-release. Hence, dissolution of the silica matrix can be discarded as the main factor in the release of the encapsulated CBD, even though some dissolution does occur.

**Figure 5:**
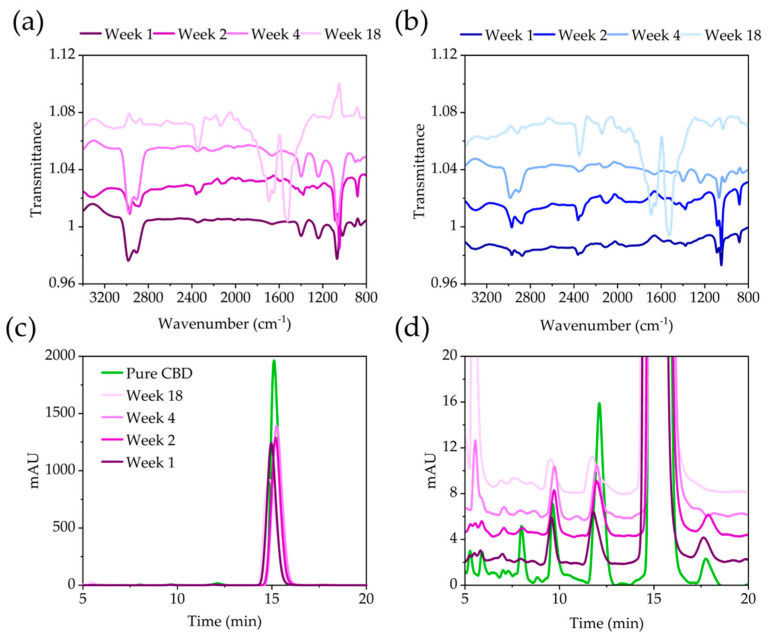
Stability of CBD extracted from PVA-CBD-Silica particles after prolonged storage. FT-IR spectra of samples stored at (**a**) 40 °C and 60% humidity and (**b**) 4 °C for up to 18 weeks. (**c**) Chromatogram of samples stored at 40 °C and 60% humidity compared to that of pure CBD, and (**d**) an inset showing the lower intensity peaks. Samples have been stacked for ease of viewing.

The stability of pure and encapsulated CBD was assessed, as this is a critical parameter in cannabinoid stability [14], at 4 °C, as well as 40 °C with 60% humidity to accelerate degradation. During the 18-week period, small strips of the PVA-CBD-Silica were cut and the CBD extracted in ethanol, as previously described. FT-IR and HPLC measurements were conducted to assess any changes to the CBD. Figure 5a,b shows the FT-IR spectra of PVA-CBD-Silica samples at 4 °C, as well as 40 °C under rapid degradation conditions. At both temperatures, the extracted CBD from the PVA matrix shows a significant change in the main bands between 2800–3100 cm^−1^, 1400–1800 cm^−1^ and at 1060 cm^−1^ during the time studied [27]. There is a loss in the strength of the vinyl stretching band at 3070 cm^−1^ after 1 week of storage at both temperatures, which is observed in previous studies [35]. The stability of the (C–C–H) aliphatic moiety (1360 cm^−1^) and the aromatic C–OH vibration (1200 cm^−1^) is preserved for a longer time at 4 °C than at 40 °C with 60% humidity. After 18 weeks, CBD extracted from PVA-CBD-silica films showed considerable degradation, with strong bands at 2335 cm^−1^ and between 1500–1800 cm^−1^. Spectra for the degradation of pure CBD are shown in Figure S4a–c and show similar degradation to the CBD extracted from PVA-Silica after 18 weeks of storage. The strong band at 2335 cm^−1^ can be assigned to the asymmetric stretch of carbon dioxide (O=C=O). The absence of any antisymmetric and symmetric (C–O–C) stretching bands, however, excludes the formation of any THC or CBN derivative during the degradation process [27]. Despite the apparent changes to the IR spectra, HPLC measurements for CBD extracted from PVA-CBD-Silica samples show the characteristic retention peak at 15 min (Figure 5c,d), albeit with decreased intensity, and the presence of additional minor peaks at lower retention times, which were also found in the pure CBD and are likely due to the presence of small traces of cannabinoid impurities [36]. Chromatograms obtained from the CBD extracted from PVA-CBD-Silica show minor peaks characterized by a splitting of the peak at 12 min, originally observed for the pure CBD; the formation of a new peak at 6 min; and the disappearance of the peak at 17 min with prolonged storage time. This indicates that some degradation, albeit small, is occurring. When pure CBD is degraded under similar conditions, its HPLC trace shows the formation of a sharp and intense peak at 5.3 min after week 4 of storage, accompanied by the disappearance of the peak at 17 min (Figure S5).

### 3.2. Ex Vivo Transdermal Studies

**Figure 6:**
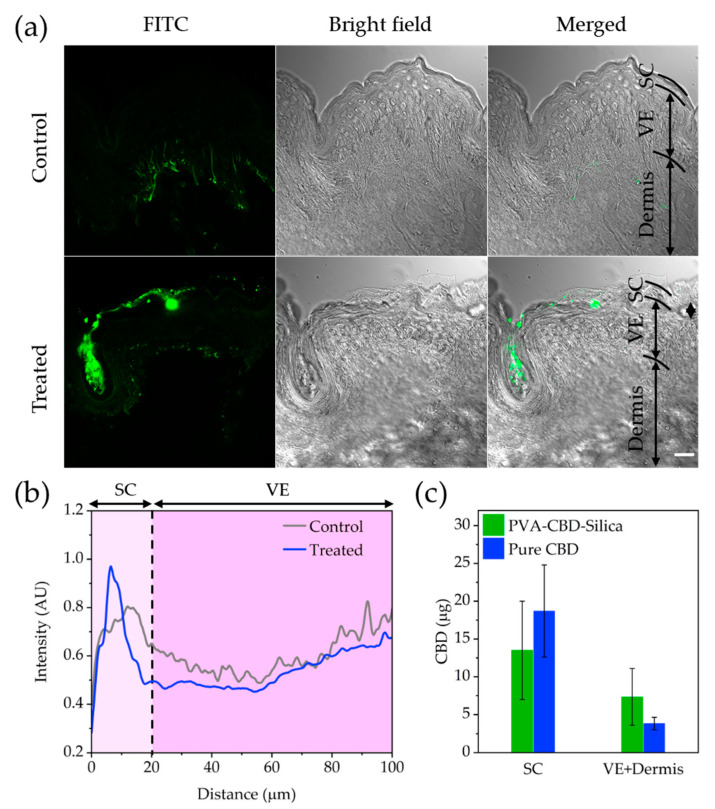
(**a**) Human skin penetration of PVA-FITC-Silica. LSCM imaging of cross-sectioned untreated control (first panel) and treated human skin with PVA-FITC-Silica (second panel) after 72 h exposure. Left, middle and right columns show green pseudo-coloured (FITC), bright-field and merged microscopy channels, respectively. Scale bar, 20 μm. (**b**) Penetration profiles of PVA-FITC-silica in control and treated human skin. (**c**) Total CBD concentrations measured in different layers of human skin treated with either pure CBD or PVA-CBD-silica. The stratum corneum, viable epidermis and dermis layers of skin are designated as SC, VE and Dermis, respectively.

LSCM imaging was used to visualize the transdermal penetration of FITC-Silica particles released from PVA film within human skin (PVA-FITC-Silica; see Materials and Methods). Then, the microscopy images were analysed with ImageJ software to obtain the fluorescence intensity profile or penetration profile of the FITC-Silica particles as a function of skin depth (distance from skin surface). Figure 6a shows confocal microscopy images of untreated (control) and treated human skin with PVA-FITC-Silica films. It is evident that FITC-Silica particles released from PVA film accumulate in superficial layers of SC, with no further penetration into the viable epidermis (VE) layer (Figure 6b). The fluorescence signal in the VE layer was found to be comparable with the endogenous fluorescent signal of skin tissue. Consistent with the microscopy observations, the HPLC data shown in Figure 6c revealed that the detected CBD in the SC layer of skin treated by either pure CBD or PVA-CBD-silica was higher than the CBD recovered from the VE and dermis. While the CBD levels in the SC layer of pure CBD-treated samples were approximately 38% higher than the SC of PVA-CBD-silica treated samples, the CBD measured in the VE and dermis layers was twofold higher for the PVA-CBD-Silica than the pure CBD-treated samples. No trace of CBD was detected in the receptor fluid within the Franz cells for any of the samples. These results are not surprising, as highly lipophilic compounds, such as CBD, can have a higher affinity to SC than the hydrophilic VE and dermis layers, which limits their penetration [37]. However, trapped organosilica particles in SC and hair follicles can act as a “depot” that favours prolonged and sustained release of CBD into viable layers of skin.

## 4. Discussion

In this study, we characterize CBD encapsulated within an organosilane matrix (CBD-Silica), with a loading amount of 26.4 wt%. Daniels et al. [38] designed mesoporous silica (Aeroperl 300) CBD formulations for buccal delivery, where the maximum loading capacity of CBD was 16.4 wt%. The use of an organosilane matrix containing phenyl groups, together with a direct loading procedure of the CBD conducted under room-temperature conditions, results in a relatively high loading capacity of the stable compound. The CBD-Silica particles are easily embedded within a thin PVA film, capable of releasing stable CBD with an enhanced dissolution profile, in comparison to the CBD-Silica particles alone and pure CBD. The solubility enhancement is consistent with the prevailing mechanism of mesoporous encapsulation and drug release, involving the stabilization of an amorphous state of the drug with the mesopores. However, this effect is not as dramatic as that observed for other compounds, where the solubility is more dependent on crystallization behaviour or pH [39,40]. In the case of the organosilane particles presented here, evidence for microporosity is observed upon calcination of the particles (surface area 288 m^2^/g), indicating that the CBD occupies nanoscale pockets created during the direct loading of the compound. The fastest release was observed in simulated sweat, with approximately 9% of the loaded CBD released in the first hour, and over 50% in 24 h. This is considerably slower than values obtained by Daniels et al. in artificial saliva, where 100% release was detected after 1 h [38]. Their experiments were conducted at a faster stirring speed of 65 rpm, and it must be noted that the initial dissolution of the PVA results in a delay in CBD release from the organosilica particles of between 10–60 min (Figure S3), especially with simulated intestine media.

The stability of released CBD was measured over prolonged times after storage of the PVA-CBD-Silica and pure CBD at 4 °C or 40 °C (and high humidity), with only a difference in the degradation behaviour overall. However, the formation of small levels of degradation products can be detected in the chromatograms of the released CBD from the PVA-SBD-Silica formulation (Figure 5d) after 4 weeks of storage at 40 °C and 60% humidity, which is also accompanied by a decrease in the main retention peak. Further work is required to identify these small impurities detected in our study; however, these constitute a small proportion of the remaining CBD formulation, at time periods that are considerably longer than the predicted 3-day shelf-life (t_90%_) of CBD resin at 25 °C [41]. Of significance to the application of the product is the confirmation that no transformation to THC was observed from FT-IR spectroscopy. The main degradation product observed in the chromatograms of pure CBD at similar time points was not observed in the CBD obtained from PVA-CBD-Silica (Figure S5).

This depot release from particles penetrating towards the lower part of the SC has been demonstrated previously with mesoporous silica nanoparticles using transdermal enhancers [42]. Ex vivo transdermal studies conducted here show that whilst the organosilica particles do not cross the stratum corneum (SC) layers of the skin, the silica particles do remain there after 72 h of the experiment (Figure 5), thus enabling the release of the CBD cargo. The CBD measured in viable epidermis (VE) and dermis layers was higher overall for the PVA-CBD-silica than the pure CBD-treated sample, consistent with an enhancement in the dissolution profile. The concentration of CBD detected in VE and dermis layers was 7.33 μg ± 3.7, which is equivalent to only 0.41% of the total amount of CBD loaded in the PVA formulation. This was higher than that obtained for pure CBD (0.27%). Stinchcombe et al. reported a transdermal synthetic cannabinoid patch capable of delivering a cumulative amount of 22.9 ± 1.2 μg per cm^2^ of human skin after 48 h [43]. This led to an in vivo, mean steady-state concentration of 8.6 ng/mL for the same formulation in plasma from a Guinea pig model. In comparison, the transdermal CBD formulation described here for CBD obtained a concentration of 5.55 μg per cm^2^ of skin after 72 h. Hence, a slower release kinetics may be expected from the PVA formulation presented here. It is important to note that these concentrations are below those expected to give a therapeutic effect for CBD in plasma, 50 ng/mL, which has been estimated from the concentration most frequently found in the plasma of patients with refractory epilepsy receiving oral doses ranging from 0.05 to 9 mg/kg/day [44]. Thus, optimization of the dose and further enhancement of the transdermal penetration are required for the formulation to be successfully implemented.

Further studies are required to understand the role of PVA in the release and penetration of CBD and other cannabinoids, and if the dose loaded within the organosilica particles can reach sufficient plasma levels for therapeutic purposes [10,12]. Our work highlights the challenges posed by transdermal penetration of highly lipophilic compounds, where barriers, such as solubility and penetration across the stratum corneum, need to be overcome.

## 5. Conclusions

A silica nanoparticle-based formulation is presented as capable of stabilizing CBD for prolonged time periods of up to 18 weeks, with only small levels of degradation products observed by HPLC. No degradation of CBD to the psychedelic compound THC is observed. In vitro dissolution experiments support a mechanism of solubility enhancement for CBD, which is more prominent in simulated sweat media, as the compound is released from the particle matrix. Transdermal penetration studies show the presence of CBD within the dermis layer of the skin at below therapeutically relevant concentrations. However, the concentration of CBD measured below the stratum corneum was higher for PVA films containing the encapsulated compound than for pure CBD. The role of PVA as a potential enhancer should be investigated in further studies.

These results highlight the difficulty in delivering hydrophobic compounds using transdermal patch formulations, but further optimization of PVA films, as well as the loading concentrations, should yield therapeutic concentrations of CBD through skin.

## Data Availability

The data presented in this study are available on request from the corresponding author.

## Supplementary Materials

The following supporting information can be downloaded at: https://www.mdpi.com/article/10.3390/pharmaceutics15030798/s1—Figure S1: XRD analysis of the organosilica particles in their dried, calcined and extracted state; Figure S2: Thermogravimetric analysis of as received CBD-silica sample; Table S1: Elemental Microanalysis (CHN analysis) of CBD-silica sample; Table S2: Components of the dissolution media; Figure S3: Release experiments in simulated fluids for CBD formulations; Figure S4: FT-IR of pure CBD and CBD extracted from the PVA-CBD-Silica; Figure S5: HPLC peaks for pure CBD store at 40 °C and 60% humidity.
