# Supplementary material for: Enhanced Skin Penetration of Cannabidiol Using Organosilane Particles as Transdermal Delivery Vehicles
Source: Pharmaceutics. 2023 Feb 28;15(3):798. doi: 10.3390/pharmaceutics15030798 (PMC10057149; doi:10.3390/pharmaceutics15030798)
Supplement: Supplementary file 1 [file pharmaceutics-15-00798-s001.zip › pharmaceutics-2226561-supplementary.pdf]

Supporting Information

Enhanced Skin Penetration of Cannabidiol Using Organosilane Particles as Transdermal Delivery Vehicles

Zahra Khabir, Connie Partalis, Jimit Vijay Panchal, Anand Deva, Aparajita Khatri and Alfonso Garcia-Bennett *

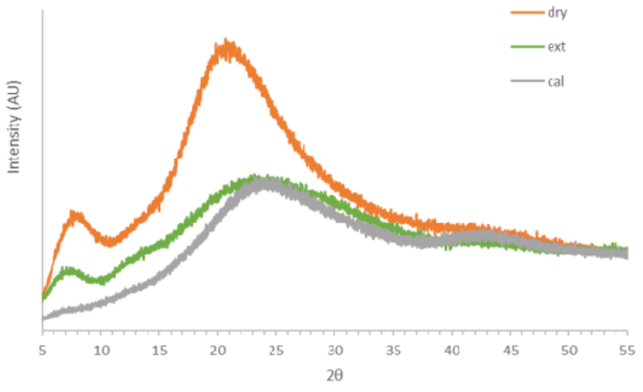

Figure S1. XRD analysis of the organosilica particles in their dried, calcined and extracted state with intensity of 2Θ, Cu-Kα radiation as X-Ray source.

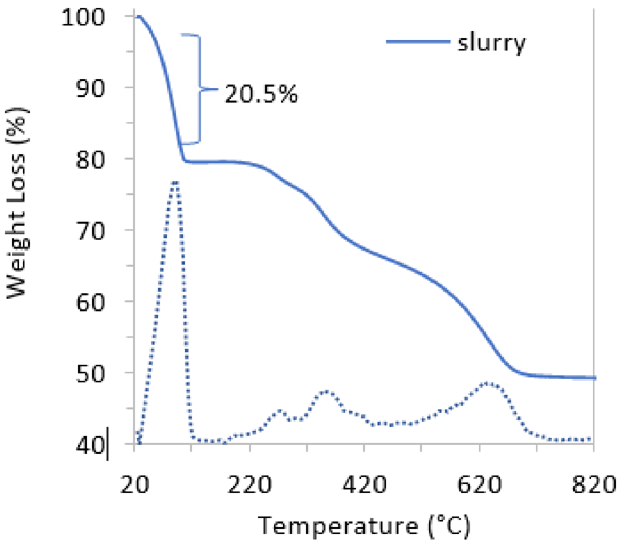

Figure S2. Thermogravimetric analysis of slurry CBD-Silica particles, containing 20wt% water. Doted lines correspond to the first derivative curves of the weight loss curves shown. Slurry samples show four distinct weight loss regions in, owing to; (I) removal of physically adsorbed water at 100 °C, (II) de-sorption of propylamine groups at 220-320 °C

corresponding to 3.1 wt%, (III) decomposition of CBD between 320-420 °C corresponding to a weigh loss of 9.7%, and (IV) decomposition of phenyl groups at 500-700 °C which is 17.2wt%.

Table S1. Elemental Microanalysis (CHN analysis) of CBD-Silica particles. Values are expressed as grams of element per 100 grams of sample. For organic standard materials the trueness 95% confidence limit of the technique is ± 0.3% with a precision of ± 0.2%. Analysis conducted after complete extraction of the CBD.

**Sample**	**C%**	**H%**	**N%**
Slurry	39.07	4.648	3.49
Extracted	48.32	5.978	2.54
Dried	30.25	7.7	1.51

Table S2. Components of the dissolution media.

Name of the fluids	Components (for 2L)	pH	Solubility of CBD (after 48 hours)
SIF	13.61g potassium phosphate monobasic and sodium hydroxide to reach pH	6.8	> 10 %
SGF	4g sodium chloride and hydrochloric acid to reach pH	1.2	> 10 %
SSW	10g sodium chloride, 1g urea, 2ml lactic acid and ammonium hydroxide to reach pH	6.6	< 50 %

SIF – Simulated intestine fluid; SGF – Simulated gastric fluid; SSW – Simulated sweat.

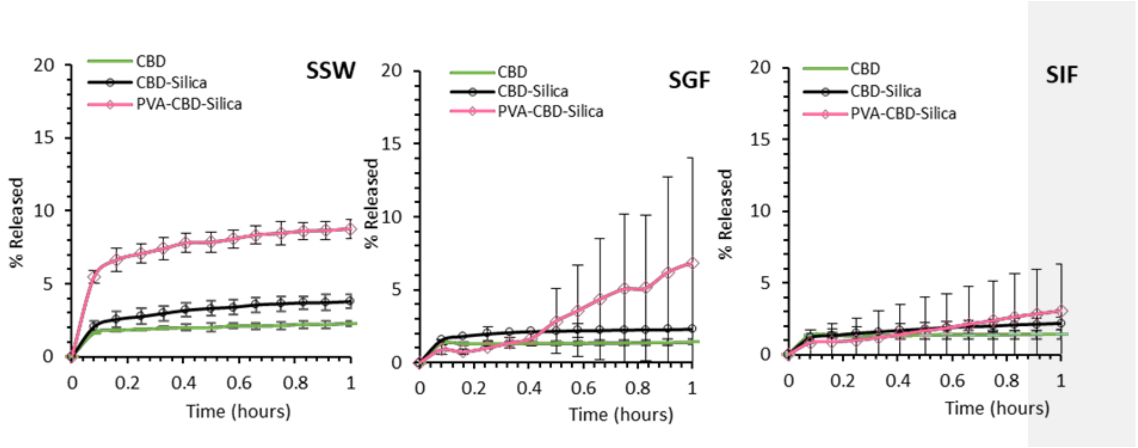

Figure S3. Release experiments in simulated fluids for CBD formulations showing the relative differences in the solubility of the PVA film (as a function of CBD release) in the first hour of contact in media.

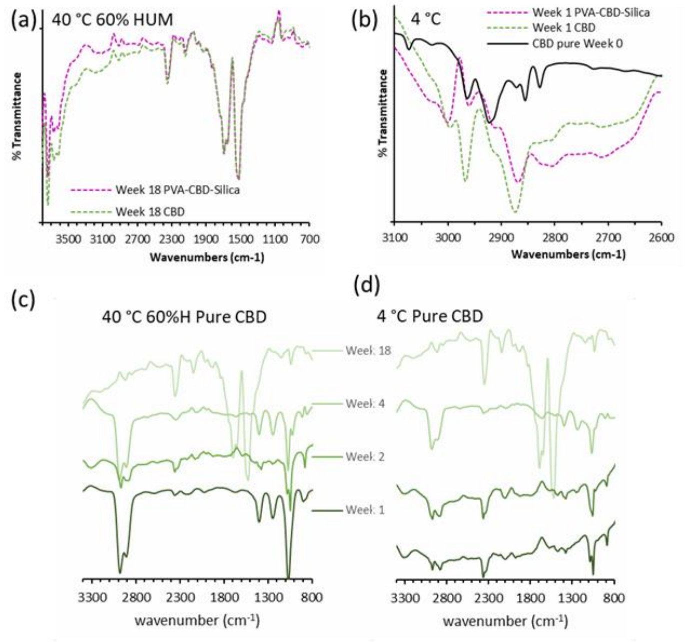

Figure S4. (a) FT-IR of Week 18 samples of pure CBD and CBD extracted from the PVA-CBD-Silica. (b) FT-IR of pure CBD and CBD extracted from the PVA-CBD-Silica at week 1 of storage in 4°C highlighting the loss of the peak at 3070 cm^{-1}. For comparison pure CBD at week 0 is also shown. (c) Time dependence of the FT-IR spectra of pure CBD at 4 °C and 40 °C 60% humidity.

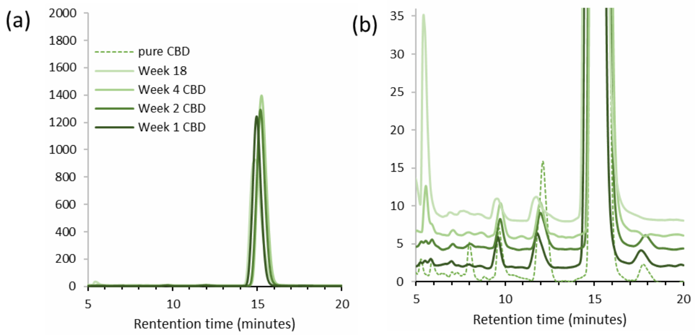

Figure S5. (a) HPLC peaks for pure CBD store at 40 °C and 60% humidity. (b) the inset shows the formation of additional peaks after Week 1 which remain relatively constant throughout the degradation period. After Week 4 an additional peal at 5 mins is seen, which increases significantly at week 18.
